# Identification of a Novel Interferon-Stimulated (ISG15) Gene Variant Associated With Inflammatory Cutaneous Lesions and Zinc Deficiency in a Unique Family: A Case Series and Literature Review

**DOI:** 10.7759/cureus.50701

**Published:** 2023-12-17

**Authors:** Ali Yahya B Alzahrani, Linah Saleh Abbas Alghamdi, Fahad A Alghamdi

**Affiliations:** 1 Paediatrics and Child Health, King Fahad General Hospital, Jeddah, SAU; 2 Radiology, King Fahad General Hospital, Jeddah, SAU

**Keywords:** dermatological manifestations, zinc deficiency, inflammatory cutaneous lesions, interferon-stimulated gene 15, isg15

## Abstract

Interferon-stimulated gene 15 (ISG15) is a pivotal protein involved in antiviral defense and immune regulation. This study presents a remarkable case series of a consanguineous family with a homozygous variant in the ISG15 gene, leading to a complex interplay of intriguing dermatological manifestations and concurrent zinc deficiency. The range of cutaneous phenotypes observed in the family members, from severe ulcerative lesions to atopic dermatitis, highlights the intricate relationship between the identified genetic variant and dermatological conditions. Furthermore, zinc deficiency adds another layer of complexity to the understanding of these conditions. Comprehensive assessments of zinc levels were conducted for three siblings, while the fourth sibling's evaluation was impeded. This extraordinary case series offers a unique opportunity for scientific exploration, shedding light on complex genetic disorders and potentially paving the way for novel diagnostic and therapeutic strategies in medical science. The convergence of familial genetics, the homozygous ISG15 variant, and the captivating spectrum of cutaneous manifestations hold promise for advancing our understanding of these conditions and their underlying mechanisms.

## Introduction

Interferon-stimulated gene 15 (ISG15) is a pioneering ubiquitin-like modifier protein involved in protein conjugation, known as ISGylation [1]. It plays a crucial role in antiviral defense and is highly induced following stimulation by type I interferons or pathogenic infections [1,2]. The secreted form of ISG15 in humans exerts potent immunomodulatory effects, including promoting cytokine production and enhancing the proliferation of natural killer (NK) cells [3]. In addition, ISG15 is primarily stored in neutrophils and selectively released during bacterial challenges, highlighting its involvement in host defense against microbial pathogens [2,3]. ISG15 is also actively secreted by various cell types and acts as a robust cytokine, inducing interferon gamma (IFN-γ) production in lymphocytes, particularly NK cells [4]. The interplay between ISG15 and immune mediators like interleukin-12 (IL-12) synergistically enhances the immune response against viral infections [3-5]. Mutations in ISG15 have been associated with compromised IFN-γ-mediated immunity, rendering individuals more vulnerable to mycobacterial infections, as seen in Mendelian susceptibility to mycobacterial diseases (MSMD) [4,5].

In this study, we present a groundbreaking case series involving a consanguineous family. The parents, both carriers of a genetic variant, have four remarkable offspring, two males and two females, who share a homozygous variant of uncertain significance within the ISG15 gene. Pathogenic variants in the ISG15 gene have been unequivocally linked to autosomal recessive immunodeficiency type 38. What sets this case series apart is the intricate interplay between the identified genetic variant and a diverse spectrum of intriguing dermatological manifestations observed in these family members. The range of cutaneous phenotypes, from severe ulcerative lesions to the complexities of atopic dermatitis, is captivating and perplexing. Adding another layer of complexity, three out of the four siblings also exhibit concurrent zinc deficiency, further confounding the understanding of the underlying dermatological conditions. While comprehensive assessments of zinc levels were conducted for three of the siblings, personal issues impeded the evaluation of the fourth sibling's zinc status, introducing an element of uncertainty.

This extraordinary case series, encompassing familial genetics, the enigmatic homozygous variant in the ISG15 gene, and the captivating spectrum of cutaneous manifestations, provides a unique opportunity for scientific exploration. The convergence of these factors holds tremendous promise for advancing our understanding of complex genetic disorders and may pave the way for novel diagnostic and therapeutic strategies in the field of medical science.

## Case presentation

We present a case of a girl born in 2012 from a consanguineous family. Shortly after birth, the patient received the bacillus Calmette-Guérin (BCG) vaccination, which elicited an exaggerated response characterized by left axillary lymphadenopathy and erythema of the overlying skin, accompanied by the discharge of a purulent material. At the age of one month, the patient presented with ulcerated lesions in the perianal region. Hospitalization ensued, but antibiotic treatment failed to yield a clinical response.

However, after two months, the lesions spontaneously resolved. Subsequently, the patient was transferred to a specialized medical center, where a diagnosis of zinc deficiency was established. Oral zinc supplementation was initiated at the age of one month. However, at three years of age, new lesions emerged in the inguinal region proximal to the external genitalia, exhibiting bilateral and asymmetrical distribution. Additionally, lesions appeared on the left axilla, as well as the anterior and posterior aspects of the neck, despite both local and systemic therapeutic interventions. Partial healing of the lesions was observed after six months, but new ulcers subsequently emerged on the back of the neck and in both inguinal regions. These newly formed ulcers displayed increased size, significant crustations, and severe discharge, accompanied by infected necrotic skin lesions in the left axilla.

Multiple investigations were conducted to elucidate the underlying etiology. Notably, various diagnostic tests, including periodic acid-Schiff, acid-fast bacilli, and Ziehl-Neelsen reactions, yielded negative results. Wound cultures performed three times revealed the presence of pseudomonas with fungal infection, abundant fungal infection, *Enterobacter cloacae*, and methicillin-resistant *Staphylococcus epidermitis*, respectively. A biopsy of the left axillary lesions showed necrotic subcutaneous tissues with evidence of inflammatory cell infiltrate and congestion.

**Figure 1:**
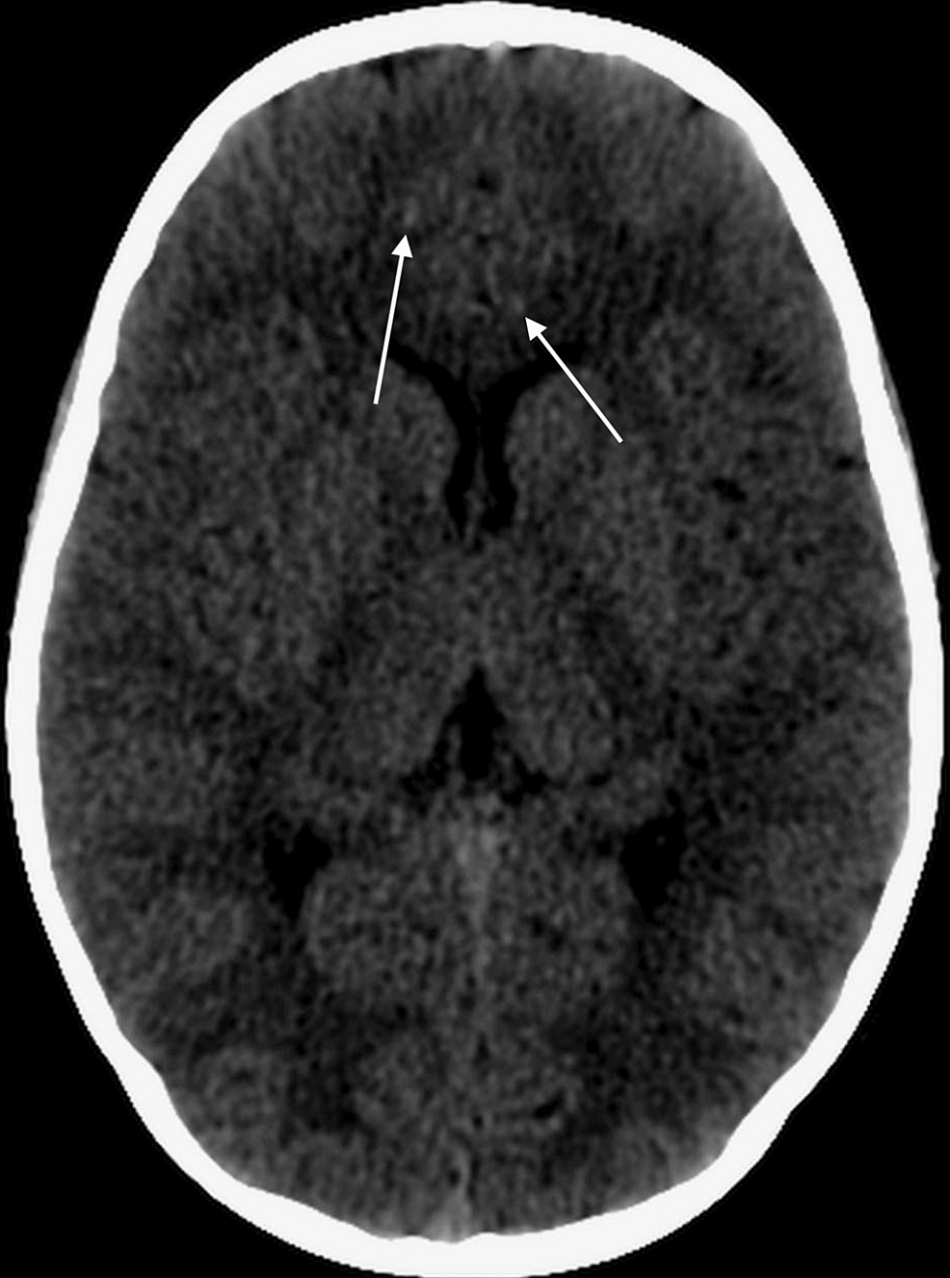
The brain computed tomography (CT) examination demonstrates the presence of discrete bilateral subcortical focal areas exhibiting subtle hyperdensity within the frontal lobes of the brain. These focal regions exhibit discreet calcific deposits, giving rise to localized enhancements in radiodensity within the cerebral parenchyma.

Despite prolonged courses of broad-spectrum antibiotics, the patient's condition failed to improve and, in fact, exhibited a worsening course. Further investigations revealed persistent leukopenia and positive cytomegalovirus and rubella IgG, suggesting an ongoing immunological disturbance. Whole exome sequencing (WES) was subsequently performed, revealing the identification of a homozygous variant of uncertain significance within the ISG15 gene. Pathogenic variants within this gene have been associated with autosomal recessive immunodeficiency type 38 with variant coordinates NM_005101.3:c.463dup. In addition, a computed tomography (CT) scan of the brain demonstrated the presence of intracranial calcifications (Figure 1).

To address the patient's complex condition, oral prednisone was initiated with a carefully planned tapering regimen, aiming to modulate the immune response and mitigate the pathological processes associated with the disease. The comprehensive nature of this clinical case underscores the intricate interplay between genetic factors, immunodeficiency, zinc deficiency, and the complex array of cutaneous manifestations observed in this patient.

In this captivating case series involving a consanguineous family, three additional siblings have been identified with intriguing medical presentations and shared genetic defects. The second boy, born in 2009, presented with cutaneous lesions resembling those observed in the first sibling. Following a comprehensive evaluation, he was also diagnosed with zinc deficiency, a condition known to impact various cellular processes, including immune function and skin health. Similarly, the third sibling, a girl born in 2006, exhibited skin lesions reminiscent of those observed in the first and second siblings. However, the severity of her dermatological manifestations surpassed that of her siblings, suggesting a potentially more aggressive disease course. Consistent with the pattern observed in the other affected siblings, she, too, was diagnosed with zinc deficiency. Consequently, all of them were initiated on zinc supplementation to address the underlying deficiency.

The rationale behind this intervention was to rectify the observed zinc deficiency and its potential impact on immune function and skin health, which may contribute to the exacerbation of the dermatological phenotypes. The fourth sibling, a boy born in 2005, presented with atopic dermatitis characterized by intense pruritus, erythematous, and eczematous skin lesions. However, he did not undergo zinc level assessments due to personal circumstances.

The management approach for this sibling involved the use of topical steroids, which serve to mitigate inflammation, alleviate symptoms, and restore the integrity of the skin barrier. All siblings in this case series have not experienced any intensive care unit admissions since birth and have a normal developmental history.

## Discussion

The discovery of pathogenic variants within the ISG15 gene has emerged as a recent and significant breakthrough, establishing it as a distinct etiology of type I interferonopathy in the human population [6,7]. Initially, individuals identified as having immunodeficiency 38 (Online Mendelian Inheritance in Man (OMIM): 616126), an autosomal recessive condition linked to syndromic MSMD [7], were found to harbor biallelic loss-of-function variants in the ISG15 gene. Remarkably, despite the well-documented antiviral role of ISG15 in murine models, these patients did not manifest the anticipated viral susceptibility phenotype [5-8]. Consistently, individuals exhibiting biallelic pathogenic variants in ISG15 consistently showcase heightened ISG expression and display cerebral calcifications, radiologically resembling Aicardi-Goutières syndrome (AGS) [9].

In our study, we employed WES to diagnose immune-related disorders in a consanguineous Saudi family, leading to an expanded phenotype associated with ISG15 deficiency. The newly identified features include zinc deficiency and cutaneous inflammatory manifestations, which occurred independently of mycobacterial infections. This highlights the immunomodulating role of ISG15, as a homozygous variant of uncertain significance was found in the ISG15 gene, known to be associated with autosomal recessive immunodeficiency type 38. Notably, brain CT scans revealed intracranial calcifications in multiple patients, and high levels of ISG products were detected in their blood samples [10]. Moreover, the presence of ISG15 deficiency further substantiates its classification as a genuine type I interferonopathy [2,6].

While inflammation of the skin is not uncommon in interferonopathies type I, a previous study had reported distinct skin lesions in individuals with ISG15 deficiency, setting them apart from the cold-induced inflammatory conditions observed in familial AGS [4,7]. The distribution pattern of skin lesions in ISG15-deficient patients was exceptional, primarily affecting regions, such as the scalp, neck, axillary, perineal, and groin areas [8]. By contrast, patients with AGS carrying the TREX1 D18N mutation exhibited skin lesions predominantly affecting the distal extremities, thus demonstrating a discernible pattern of skin inflammation [7-9]. The patients with ISG15 deficiency observed to have skin lesions are likely a result of complex pathophysiology [5]. Studies conducted in controlled laboratory environments, specifically in vitro and ex vivo settings, have provided evidence indicating that keratinocytes exhibit increased levels of phosphorylated signal transducer and activator of transcription 1 (pSTAT1) when ISG15 is absent. Similarly, investigations using cultured models of endothelial cells and actual tissue samples have shown that ISG15 plays a crucial role in regulating interferon-I (IFN-I)-mediated inflammation in endothelial cells [10]. In addition, the presence of CD68+pSTAT1+ macrophages in the skin lesions suggests the involvement of myeloid cells in the manifestation of these pathological skin features [6].

It is important to note that ISG15-deficient patients did not present with hemoglobinemia, arthritis, Raynaud's syndrome, post-inflammatory onychodystrophy, and systemic lupus erythematosus (SLE) resembling AGS patients with the TREX1 D18N mutation [7]. Recognizing the diverse phenotypic manifestations of ISG15 deficiency and other type I interferonopathies is of utmost importance. The variable clinical presentations, including reactions to bacille Calmette-Guerin (BCG) vaccination, intermittent seizures, and dermatological features, highlight the significance of understanding these conditions. Such understanding is facilitated by comprehending the complex interplay observed between keratinocytes, endothelial cells, and myeloid cells in IFN-I-mediated inflammation in the skin [1,3].

The cooperative interactions among neurons, astrocytes, oligodendrocytes, microglial cells, and infiltrating leukocytes within the brain offer valuable insights into the elusive pathogenesis of intracranial calcifications specifically localized in the basal ganglia [6-9]. These calcifications are believed to involve intricate cellular crosstalk and are commonly observed in pediatric patients [5]. It is of utmost importance for specialists in pediatric infectious diseases, neurology, and dermatology to maintain awareness of the diverse clinical presentations associated with these conditions. Such awareness will undoubtedly contribute to an enhanced understanding and more effective management of these complex disorders. 

Literature review

A comprehensive review was undertaken to identify pertinent scholarly publications, resulting in the retrieval of a total of 11 relevant scholarly articles. After careful evaluation, nine articles were excluded as they focused on genes other than ISG15, leaving two articles that described six individuals with ISG15 mutations associated with dermatological manifestations [1,2]. These cases, including our reported patients, represent the first documented cases worldwide of ISG15 gene mutations associated with inflammatory cutaneous lesions and zinc deficiency.

In conclusion, among the genetic forms of type I interferonopathy, ISG15deficiency stands out as functionally and clinically unique, with the exception of USP18 and STAT2 deficiencies. Its distinguishing features, including MSMD, specific patterns of dermatological lesions, and intracranial calcifications, independently contribute to its diagnostic significance. These characteristic manifestations exhibit a co-dominant inheritance pattern, setting ISG15 deficiency apart from other genetic forms of type I interferonopathy. However, the observed genotype-phenotype association should be interpreted with caution due to the analysis being based on a single family. The molecular mechanisms underlying the disease and the triggering factors for the inflammatory manifestations require further investigation.

## Conclusions

This case series of a consanguineous family and their homozygous variant within the ISG15 gene reveals the intricate interplay among genetics, immunodeficiency, cutaneous manifestations, and zinc deficiency. It sends a resounding message to medical researchers, urging them to delve deeper into the underlying mechanisms of genetic disorders and their diverse clinical presentations. By unraveling the intricate pathways involved, exploring biomarkers, elucidating disease mechanisms, and identifying therapeutic targets, researchers have unprecedented opportunities to advance our understanding of complex genetic disorders. This case series serves as a catalyst, inspiring researchers to embark on transformative investigations that hold the potential to significantly enhance patient care and pave the way for personalized treatments in the future.
